# Enhanced Energy Storage Performance through Controlled Composition and Synthesis of 3D Mixed Metal-Oxide Microspheres

**DOI:** 10.3390/nano14100825

**Published:** 2024-05-08

**Authors:** Chongjie Su, Muhammad Hilal, Fan Yang, Xinda Xu, Chao Zhang, Shuoyu Guo, Junning Zhang, Zhicheng Cai, Huimin Yuan, Wanfeng Xie

**Affiliations:** 1 College of Electronics and Information, University-Industry Joint Center for Ocean Observation and Broadband Communication, Qingdao University, Qingdao 266071, China; cjsu823@126.com (C.S.); yangfan8053@163.com (F.Y.); xdxu2022@163.com (X.X.); zc1746308456@163.com (C.Z.); guoshuoyu789@163.com (S.G.); junningzhang1@126.com (J.Z.); 2 Department of Semiconductor Systems Engineering, Sejong University, Seoul 05006, Republic of Korea; cai1121@sejong.ac.kr; 3 College of Physics and Electronic Engineering, Qilu Normal University, Ji’nan 250200, China; 4 Department of Physics, Dongguk University, Seoul 04620, Republic of Korea

**Keywords:** binary transition metal oxides microspheres, hydrothermal synthesis, aqueous asymmetric supercapacitors, specific energy, energy storage materials

## Abstract

Binary transition metal oxide complexes (BTMOCs) in three-dimensional (3D) layered structures show great promise as electrodes for supercapacitors (SCs) due to their diverse oxidation states, which contribute to high specific capacitance. However, the synthesis of BTMOCs with 3D structures remains challenging yet crucial for their application. In this study, we present a novel approach utilizing a single-step hydrothermal technique to fabricate flower-shaped microspheres composed of a NiCo-based complex. Each microsphere consists of nanosheets with a mesoporous structure, enhancing the specific surface area to 23.66 m^2^ g^−1^ and facilitating efficient redox reactions. When employed as the working electrode for supercapacitors, the composite exhibits remarkable specific capacitance, achieving 888.8 F g^−1^ at 1 A g^−1^. Furthermore, it demonstrates notable electrochemical stability, retaining 52.08% capacitance after 10,000 cycles, and offers a high-power density of 225 W·kg^−1^, along with an energy density of 25 Wh·kg^−1^, showcasing its potential for energy storage applications. Additionally, an aqueous asymmetric supercapacitor (ASC) was assembled using NiCo microspheres-based complex and activated carbon (AC). Remarkably, the NiCo microspheres complex/AC configuration delivers a high specific capacitance of 250 F g^−1^ at 1 A g^−1^, with a high energy density of 88 Wh kg^−1^, for a power density of 800 W kg^−1^. The ASC also exhibits excellent long-term cyclability with 69% retention over 10,000 charge–discharge cycles. Furthermore, a series of two ASC devices demonstrated the capability to power commercial blue LEDs for a duration of at least 40 s. The simplicity of the synthesis process and the exceptional performance exhibited by the developed electrode materials hold considerable promise for applications in energy storage.

## 1. Introduction

The combustion of fossil fuels and the resulting emission of pollutants have prompted scientists worldwide to seek alternative energy storage methods, such as clean electrochemical systems [1,2,3]. Extensive research and development have focused on emerging energy storage technologies, including supercapacitors [4], fuel cells [5], secondary batteries (such as Li, Na, K, and Li-S batteries) [6,7,8,9], and dual-battery systems for renewable energy generation [10]. These novel energy storage devices are gaining attention for their user-friendly nature, simplicity, high efficiency, and environmental friendliness [11].

Among the various kinds of energy storage devices, supercapacitors (SCs) have particular benefits due to their rapid charge and discharge rates [12]. Moreover, in comparison to secondary batteries, it may provide extremely high power densities; at the same time, the longer cycle stability and higher energy density are additional appealing advantages [1,2]. An essential component of SCs is the electrode material, whose composition, structure, and shape all have a direct impact on its electrochemical characteristics [3]. To ensure the optimal performance of supercapacitors, the electrode material must exhibit various essential qualities, such as a large specific surface area, high electrical conductivity, rapid ion transportation, and exceptional electrochemical stability [1].

In recent years, significant advancements have been made in the development of binary electrode materials, particularly for energy storage applications [13]. These materials, including carbon-based substances, conductive polymers, and blends of transition metals [14,15], show considerable promise for enhancing energy storage technologies. Transition metal complexes with diverse oxidation states and morphologies, such as Co-Fe, Ni-Fe, Ni-Co, Ni-Mo, and Co-Mo, play a crucial role in increasing energy density and extending discharge periods through rapid Faraday redox processes [16,17,18,19]. However, these materials often exhibit lower electrical conductivity and susceptibility to accumulation. To address these challenges, researchers have explored the formation of layered binary transition metal compounds (BTMCs) by combining transition metal compounds with other electrode materials known for exceptional electrical conductivity and high specific capacitance, such as Ni-Fe MOF [20], Ni/Co-MOF@aminated MXene [21] NiCo-MOF/MXene heterostructures [22], Ni–Cu nanocomposite-modified MXene [19], transition metal quantum dots@ graphene composite [23], and dual-metal MOF-derived Co-Ni/rGO [24]. These BTMC composites have shown remarkable results in supercapacitor applications, demonstrating enhanced energy density, capacitance retention, and stability after numerous cycles [24,25,26,27].

For example, Li et al. achieved remarkable results by fabricating CuO- and CoFe-based BTMCs composites using the electrosynthesis method, which exhibited an energy density of 1.857 mWh·cm^−3^ and a capacitance retention of 99.5% after 2000 cycles [28]. In a similar manner, Zhang et al. utilized an in situ crystallization technique to fabricate hybrid BTMCs composites consisting of NiMn-MB/MXene. The results showcased a remarkable specific capacitance of 1575 F·g^−1^ at 0.5 A·g^−1^, along with a capacitance retention rate of 90.3%, after subjecting the composites to 10,000 cycles under a condition of 5 A·g^−1^ [29]. Later on, researchers utilized a hydrothermal method to successfully synthesize NiO/Ni nanoparticles derived from Ni-MOF embedded on r-GO-based BTMC composites. These materials exhibited a favorable specific capacity of 649.22 C g^−1^ at 3 A g^−1^ and maintained 81.1% of their initial capacity value even after 5000 cycles at 20 A g^−1^ [30]. Using solvent-thermal and calcination processes, a fully encapsulated CoO-NiO (NiCo@Si_1_-C) BTMC-based composite was synthesized on SiO_2_-modified carbon nanofibers (Si_1_-C). The specific capacitance of NiCo@Si_1_-C composites reached 518.1 F g^−1^ at 0.5 A g^−1^, surpassing that of NiCo@Si_0_-C (229.9 F g^−1^ at 0.5 A g^−1^) by more than 2.25 times [31]. Moreover, using a one-step hydrothermal method, researchers recently succeeded in preparing a FeCoNi-LDH-based BTMC composite featuring cross-braided nanoneedle flowers. The ternary LDH utilized as the electrode material exhibits exceptional electrochemical properties, including an impressive specific capacitance of 2163.3 F g^−1^ at 0.5 Ag^−1^ and a favorable cycle retention rate of 93.8% after undergoing 5000 cycles at 20 Ag^−1^ [18].

Although BTMC-based composites significantly enhance supercapacitor performance, it is more effective to improve the structure and properties of primary transition metals or metal oxides (TMOs) through a facial-synthesis approach to create their complex structures (BTMOCs) rather than combining them with other electrode materials. This approach is favored due to the similar characteristics of TMs/TMOs, making their complexes easier to control in terms of final morphological architecture and electronic band structures, which are essential for various applications, including electrochemical energy storage. For example, Biswal et al. devised a novel electrochemical method to synthesize cobalt–nickel-mixed oxide, yielding a nanoporous sea sponge structure with interconnected nanosheets. This binary metal oxide exhibited excellent electrochemical behavior, achieving a capacitance of 76 F g^−1^ with 98% efficiency after 1000 cycles in a hybrid capacitor configuration [32]. Therefore, considering BTMOCs as a suitable structure compared to BTMCs-based composites, the selection and utilization of specific TMOs with exceptional electrochemical characteristics, such as cobalt oxide (Co_2_O_3_) and nickel oxide (NiO), is crucial. As the BTMOCs of these metals are rarely employed in supercapacitor applications due to the complexity of synthesizing them in 3D flower-like architectures.

All phases of cobalt oxide, including Co_2_O_3_, serve as promising electrocatalysts for various electrochemical activities, including energy storage applications [33,34,35]. They demonstrated exceptional redox activity across pairs like Co^2+^/Co^3+^ and Co^3+^/Co^4+^, alongside being electrochemically stable, cost-effective, and abundantly available in nature [36]. Additionally, they are electrochemically stable, cost-effective, and abundantly available in nature [25]. Moreover, when shaped into nanostructures such as nanoflowers, nanosheets, and nanoflakes, Co_2_O_3_ exhibits further enhanced electrochemical properties [26]. Conversely, NiO shows great potential for energy storage applications due to its ideal band-edge potential for generating redox-active O_2_^−^ and OH^−^ radicals, which enhance active sites in electrochemical reactions [26]. However, the sluggish electrochemical kinetics of NiO pose challenges for charge transfer, particularly due to slow ion storage kinetics and rapid volume changes during charging/discharging processes [37]. These challenges can be addressed by controlling crystal size and engineering NiO into three-dimensional (3D) architectures [26,38]. As crystal size affects surface area, crucial for electrochemical reactions. Meanwhile, different 3D structures, including cactus-like, spherical, hierarchical, porous, and flower-like configurations, offer increased surface areas and active sites compared to traditional nanorods, nanosheets, and nanoparticles. Thus, constructing both NiO and Co_2_O_3_ with 3D structures would be a suitable strategy to enhance their surface area and active sites, while maintaining their electrochemical stability. However, preparing both NiO and Co_2_O_3_ with a 3D flower-like architecture, even in their single form, is challenging and requires surfactants, reducing agents, and templates [39,40,41]. Additionally, the use of these additional precursors necessitates precise control over synthesis conditions, as minor changes in pressure, temperature, time, concentration, and a calcination environment can lead to different structures in the final product [42]. Therefore, developing controllable modification methods for reliably producing BTMOCs with 3D structures remains a key area of research.

In this study, layered-structured 3D BTMOCs were successfully prepared using a simple one-step synthesis method. The prepared composite consists of flower-shaped microspheres, with each nanosheet exhibiting a mesoporous structure, enhancing the specific surface area to 23.66 m^2^ g^−1^, and facilitating efficient redox reactions. Based on these characteristics, the composite demonstrated outstanding supercapacitor performances, achieving a specific capacitance of 686.4 F g^−1^ at 2 A g^−1^ and maintaining a retention rate of 52.6% after 10,000 cycles. Additionally, as an aqueous asymmetric supercapacitor (ASC) configuration, it exhibited high specific capacitance, energy density, and excellent long-term cyclability, highlighting the potential of the developed electrode materials for energy storage applications.

## 2. Materials and Methods

### 2.1. Synthesis of Flower-like CoNi Binary Micro-Balls

All reagents and chemicals were purchased from Aladdin (Shanghai, China) and utilized without additional purification. Both the pure (NiO and Co_2_O_3_) and layered BTMOCs-based samples (NiCo-95:5, NiCo-90:10, NiCo-85:15, NiCo-80:20, NiCo-75:25, and NiCo-70:30) were efficiently synthesized through a facile hydrothermal method. For instance, the preparation of NiCo-75:25 involved combining a mixed solvent (30 μL DMF and 30 μL ethylene glycol) with 12 mmol Ni(NO_3_)_2_·6H_2_O, 4 mmol Co(NO_3_)_3_·5H_2_O, and 0.6 mmol NH_4_F, denoted as solution A. Solution B was formed by dissolving 1.24 g pure terephthalic acid in 30 mL DMF and 15 mL ethanol. Subsequently, solution A was added to solution B, stirred for 1 h at 25 °C, and supplemented with 240 μL of ammonia solution. The resulting mixture was then transferred into an 80 mL Teflon-lined stainless-steel autoclave, heated at 120 °C for 12 h, and cooled to room temperature (25 °C). The product was collected, centrifuged, washed with deionized water and pure ethanol, dried for 12 h at 80 °C, and calcined for 3 h at 500 °C in a muffle furnace under ambient conditions. Following the successful synthesis of each sample, the morphological analysis and elemental composition assessment was performed using a field-emission scanning electron microscope (FESEM, Zeiss Gemini 500, Jena, Germany) and a high-resolution transmission electron microscope (HRTEM, JEM–2100 F, Tokyo, Japan) equipped with an EDS detector. The structural analysis was validated through X-ray diffraction (XRD) patterns (Bruker AXS, Billerica, MA, USA (D8, Advance, Cu K*α* X-ray source) diffractometer with Cu K*α* radiation (*λ* = 0.15418 nm)), and the XRD scanning occurred at a rate of 0.2° s^−1^ within a 2*θ* range of 10° to 80°. The spectra of X-ray photoelectron spectroscope (XPS) were recorded using a Termo Scientific Escalab 250xi instrument (Waltham, MA, USA) equipped with a monochromatic Al Kα source. Additionally, we conducted a BET analysis (BET MicroActive ASAP 2460, Tokyo, Japan) to determine the pore size and specific surface area of the BTMOCs.

### 2.2. Fabrication and Electrochemical Characterization of Supercapacitor

The slurries for each working electrode were prepared by mixing the active materials (e.g., NiCo-75:25: 80%), along with 10% PVDF and 10% carbon black, in NMP as the solvent. The resulting blends were ground using a mortar and pestle for 20 min. Coating of the slurry was then carried out on commercially available nickel foam (effective coating area = 1 × 3 cm^2^), followed by drying in an oven for 12 h. The loading mass was estimated to be 2 mg.

The NiCo-75:25-based electrode served as the working electrode for individual electrochemical assessments within a three-electrode system. A freshly prepared 3 M KOH aqueous solution was used as the electrolyte, with a saturated AgCl/Ag electrode as the reference electrode and a platinum plate as the counter electrode. The evaluations included cyclic voltammetry (CV), galvanostatic charge–discharge (GCD), and electrochemical impedance spectroscopy (EIS) measurements conducted at room temperature.

The specific capacity (Cs: F/g) was quantitatively analyzed using GCD curves and Equation (1): [43].

(1) $$C=\frac{I∆T}{M∆V}$$

where *I*/*M* represents the applied current density (A/g), Δ*V* is the voltage (V), and Δ*T* denotes the discharge time (s).

An asymmetric supercapacitor (ASC) was fabricated using NiCo-75:25 and activated carbon (AC) as the positive and negative electrodes, respectively, in a 3 M KOH electrolyte with a separator. The fabrication method for the negative electrode was identical to that of the positive electrode. The mass fraction of NiCo-75:25 to AC was determined using the charge balance formula (q^+^ = q^−^) [43].

(2) $$\frac{m_{+}}{m_{-}}=\frac{C_{-}\times ∆V_{-}}{C_{+}\times ∆V_{+}}$$

where *q*, *C*, Δ*V*, and *m* denote the electrode’s charge, capacitance, potential window, and mass. The optimal NiCo-75:25-to-AC mass ratio was approximately 1:1, with an overall weight of about 3.1 mg cm^−2^.

The energy density (ED) in Wh kg^−1^ and power density (PD) in W kg^−1^ of the ASC were calculated using specific relationships, as given in Equations (3) and (4) [44].

(3) $$E=\frac{1}{7.2}\;C_{s}\;{\left(∆V\right)}^{2}$$

(4) $$P=\frac{E\times 3600}{∆T}$$

## 3. Results and Discussion

### 3.1. Morphological Analysis

**Figure 1:**
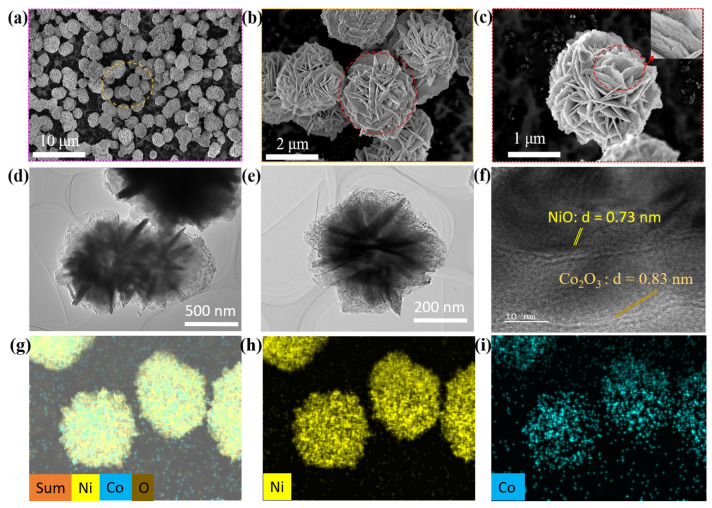
(**a**–**c**) SEM images depict various magnitudes of the NiCo-75:25 BTMOCs, with the inset image in (**c**) revealing the porous nanosheets. (**d**–**f**) Homogeneous and porous nanostructures observed through HRTEM analysis, confirming the lattice fringe spacing of NiO and Co_2_O_3_. (**g**–**i**) EDS mapping confirming the spatial distribution of Ni and Co components, along with oxygen.

The SEM analysis was conducted at various resolutions to elucidate the morphology of the BTMOCs, as depicted in Figure 1a–c. The lower magnification images (Figure 1a,b) showcase the formation of distinct flower-shaped microspheres (MSs). To delve deeper into the intricate details of the BTMOCs, higher magnification images were acquired (Figure 1c), revealing the porous structure of the nanosheets constituting each BTMOC. These nanosheets form a robust interconnected network, with their surfaces exhibiting a terrace-like structure rather than a smooth and flat appearance (inset of Figure 1c). Furthermore, the average diameter of these hierarchical BTMOCs was measured at 2.2 ± 0.2 µm.

The homogeneous and porous nature of the BTMOCs, along with the confirmation of their structure and elemental composition, was further verified through an HRTEM analysis, as depicted in Figure 1d–f. Figure 1d,e clearly illustrate the porous nanostructures of each nanosheet and the overall homogeneity of the BTMOCs. To observe the structural analysis and confirm the lattice fringe spacing, a high-resolution image was captured, as depicted in Figure 1f, indicating that the BTMOCs are composed of NiO and Co_2_O_3_, as evidenced by their lattice fringe spacing of approximately 0.73 nm and 0.83 nm, respectively. This value aligns well with the (200) and (311) planes of NiO and Co_2_O_3_, respectively, which were also observed in the XRD pattern of the BTMOCs, as discussed in subsequent sections.

Furthermore, elemental confirmation was substantiated through EDS mapping, as presented in Figure 1g–i. These images illustrate a well-matched spatial distribution of the prepared BTMOCs, consisting of a higher Ni content and lower Co content, along with oxygen.

### 3.2. Structural and Surface Analysis

**Figure 2:**
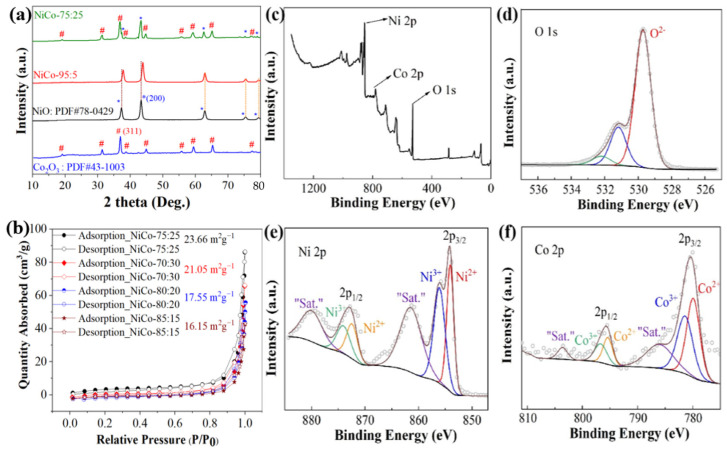
(**a**) XRD patterns and (**b**) N_2_ adsorption/desorption isotherm of the prepared BTMOCs MSs. XPS spectra of NiCo-75:25 BTMOCs: (**c**) full spectrum, (**d**) O 1s, (**e**) Ni 2p, and (**f**) Co 2p.

Figure 2a displays the XRD analysis performed to characterize the microstructure of pure Co_2_O_3_, bare NiO, Co-doped NiO composite (NiCo-95:5), and NiCo-75:25 BMTOCs MSs. The diffraction peaks of the pure Co_2_O_3_ and pure NiO samples can be readily indexed as JCPDS No. 43-1003 and JCPDS No. 78-0429, respectively [45,46]. Notably, the absence of Co_2_O_3_ peaks and the significant shift of the two major peaks towards higher diffraction angles in the XRD pattern of the NiCo-95:5 sample compared to the pristine NiO pattern indicate successful Co ion doping within the NiO structure, rather than the formation of a composite structure. Additionally, this suggests that the sample is predominantly composed of NiO due to its well-defined crystalline structure and low Co concentration. Furthermore, the XRD pattern of the NiCo-75:25 BTMOCs MSs matched well with those of NiO and Co_2_O_3_, confirming the successful preparation of the NiCo BTMOC-based composite structure. The absence of extra peaks indicates that all the samples were synthesized without impurities.

Given that the material’s surface is the primary interface with its environment, the specific surface area becomes a pivotal parameter in understanding the behavior of functional materials. In the context of supercapacitors, a substantial specific surface area offers the potential for increased electron absorption, thereby enhancing capacitor performance. To assess the specific surface area ratio and porosity of the layered BTMSs, N_2_ adsorption/desorption isotherms were employed (Figure 2b). The results indicate that the NiCo-75:25 BTMOC exhibited a notably high specific surface area of 23.66 m^2^ g^−1^, surpassing that of all other ratios used for preparing the NiCo-BTMOCs composite (Figure 2b). This characteristic is attributed to the presence of numerous voids within hierarchical nanostructures, and it aligns with the observations from the SEM and HRTEM analyses. Furthermore, the Barrett–Joyner–Halenda (BJH) analysis revealed a pore size of 20 nm, suggesting that the overall porosity of the sample primarily originates from mesopores.

**Figure 3:**
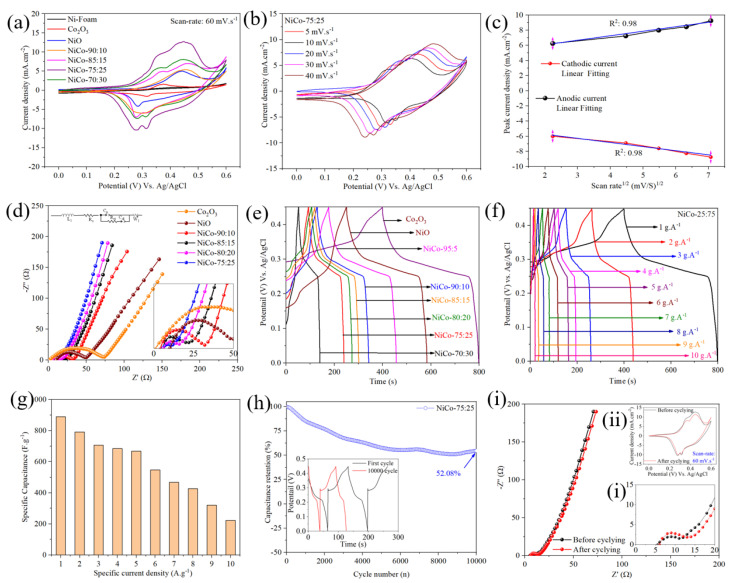
(**a**) CVs of various NiCo-based electrodes and pure Co_2_O_3_, NiO, and Ni-foam. (**b**) CVs of NiCo-75:25 electrode at different sweep rates. (**c**) Relationship between the square root of the scan rate and cathodic/anodic peak current for the NiCo-75:25 electrode. (**d**) EIS measurements comparing the solution resistance (Rs) of different electrodes, which was found by fitting with an equivalent electrical circuit (inset plot). (**e**) GCD profiles of NiCo-based electrodes and pure Co_2_O_3_, NiO at 1 A g^−1^. (**f**) GCD profiles of the NiCo-75:25 electrode at 1 A g^−1^. (**g**) Specific capacitance of the NiCo-75:25 electrode at different current densities. (**h**) Cycling stability of the NiCo-75:25 electrode over 10,000 cycles at 1 A g^−1^, with the inset plot showing the GCD at 1st and 10,000 cycles. (**i**) EIS and CV (inset plot (**ii**)) analysis of the NiCo-75:25 electrode before and after cycling stability testing.

The chemical composition and bonding states of the NiCo-5 composite were meticulously characterized through XPS analysis. The survey spectrum in Figure 3c provides a comprehensive overview of the NiCo-75:25 sample, confirming the exclusive presence of nickel (Ni), cobalt (Co), oxygen (O), and carbon (C), with no indication of any other elements. The O 1s spectra (Figure 2d) reveal two deconvoluted peaks at 529.5 and 530.7 eV, indicative of lattice oxygen (O-Ni and O-Co bonds) and chemisorbed oxygen, respectively [47,48]. Similarly, the peak observed at 532.60 eV confirm the presence of the absorbed water in the material surface [49,50]. High-resolution Ni 2p spectra (Figure 2e) exhibit peaks at 855.2 and 871.9 eV, corresponding to Ni 2p_3/2_ and 2p_1/2_, respectively [51]. The presence of the NiO phase is evident, as the Ni^2+^ and Ni^3+^ peaks contain two satellite peaks, 2p_3/2_ and 2p_1/2_, at approximately 861.6 and 878.8 eV. Similarly, the Co 2p spectrum (Figure 2f) displays two peaks at 779.7 and 794.5 eV, corresponding to the Co^2+^ valence states for Co 2p_3/2_ and Co 2p_1/2_, respectively [44]. These diverse oxidation states make both NiO and Co_2_O_3_ promising electrocatalysts for electrochemical activities. Therefore, they demonstrate exceptional redox activity across pairs like Co^2+^/Co^3+^ and Ni^2+^/Ni^4+^, while maintaining electrochemical stability due to their robust 3D structure.

### 3.3. Electrochemical and Supercapacitor Performance Analysis

The electrochemical characteristics of various electrode materials, including pure Co_2_O_3_, pure NiO, and different NiCo ratios (95:5, 90:10, 85:15, 80:20, 75:25, and 70:30), were comprehensively examined using CV, EIS, and GCD measurements, as depicted in Figure 3.

Figure 3a illustrates the CVs of different NiCo-based electrodes, along with pure Ni-foam, pure Co_2_O_3_, and pure NiO, at a constant scan rate of 60 mV s^−1^. It is observed that both NiO and Co_2_O_3_ exhibit distinct Faradaic redox peaks, representing their typical redox characteristics. The potential faradic redox reaction mechanism associated with the electrodes involves M-O/M-O-OH (M = Ni or Co ions) in the alkaline electrolyte. For instance, in the Co_2_O_3_-based electrode, a singular pair of redox peaks is distinctly observed, reflecting the reversible transformations of Co^2+^/Co^3+^ ions facilitated by anionic species (2OH^−^). These transformations can be described by the following chemical reactions: Co_2_O_3_ + H_2_O + OH^−^ → CoOOH + H_2_O + e^−^, succeeded by CoOOH + OH^−^ ⇔ CoO_2_ + H_2_O + e^−^ [52]. Similarly, the anodic and cathodic peaks in NiO arise from the oxidation of NiO to NiOOH (charging) and the reduction of NiOOH to NiO (discharging), respectively. This process follows the redox reaction, NiO + OH^−^ ↔ NiOOH + e^−^ [53,54]. Moreover, it is observed that the NiCo-90:10-based electrode exhibits a single pair of redox peaks attributed to NiO redox activity (confirming the doping of Co-ions), while NiCo-85:15, NiCo-75:25, and NiCo-70:30 electrodes display two pairs of redox peaks, indicating Co_2_O_3_ redox activity. This suggests that electrodes with Co_2_O_3_ ratios above 10% exhibit more pronounced reversible Faradic redox reactions between M–O and M–O–OH (M = Ni or Co ions) in the alkaline electrolyte [55]. Remarkably, the NiCo-75:25 electrode shows a significantly larger integrated CV area, indicating a superior capacitive behavior and enhanced electrochemical properties. Figure 3b presents CVs of the NiCo-75:25 electrode at various sweep rates (5 to 40 mV s^−1^), demonstrating slight shifts in cathodic and anodic peaks due to enhanced ion diffusion resistance at higher sweep rates [56]. However, the CV shapes remain consistent, indicating rapid redox reactions and reversible charge/discharge capabilities [56]. To further understand ion diffusion on the electrode surface, the square root of the scan rate and the cathodic/anodic peak current were plotted for the NiCo-75:25 electrode, revealing a linear relationship indicative of ion diffusion dominance over surface absorption [57] (Figure 3c).

EIS measurements (Figure 3d) show that the NiCo-75:25 electrode exhibits significantly lower solution resistance (Rs) of 1.76 Ω compared to other electrodes, owing to its optimal Ni and Co ratio, which provides abundant active sites for electrochemical reactions. Additionally, the NiCo-75:25 electrode demonstrates a shorter inclined line approaching the imaginary axis, suggesting lower total resistance and diffusion length, facilitating rapid ion diffusion into the nanostructured material [58]. The GCD profiles (Figure 3e) further confirm the superior performance of the NiCo-75:25 electrode, with the longest discharge time among all electrodes at a specific current of 1 A g^−1^, indicating the highest specific capacitance. Nonlinear GCD shapes imply Faradaic redox characteristics in all electrodes [59]. Furthermore, to quantify the Cs of the NiCo-75:25 electrode, GCD profiles were recorded at various current densities (1 to 10 A g^−1^), revealing Cs values ranging from 888.88 to 222.22 F g^−1^. The high Cs values are attributed to the abundant active sites provided by Co_2_O_3_ in the optimal ratio, facilitating rapid ion/electron diffusion during charge–discharge cycles. Long-term cycling stability tests (Figure 3h) demonstrate that the NiCo-75:25 electrode retains 52.08% of its initial capacitance after 10,000 cycles at 1 A g^−1^. Moreover, minimal variation in EIS and CV analysis (Figure 3i) before and after cyclic stability testing confirms the outstanding stability of the electrode material. These results highlight the excellent electrochemical behavior of the NiCo-75:25 electrode, positioning it as a promising material for supercapacitor applications.

### 3.4. Asymmetric Supercapacitor Performance and Real-Time Application Analysis

**Figure 4:**
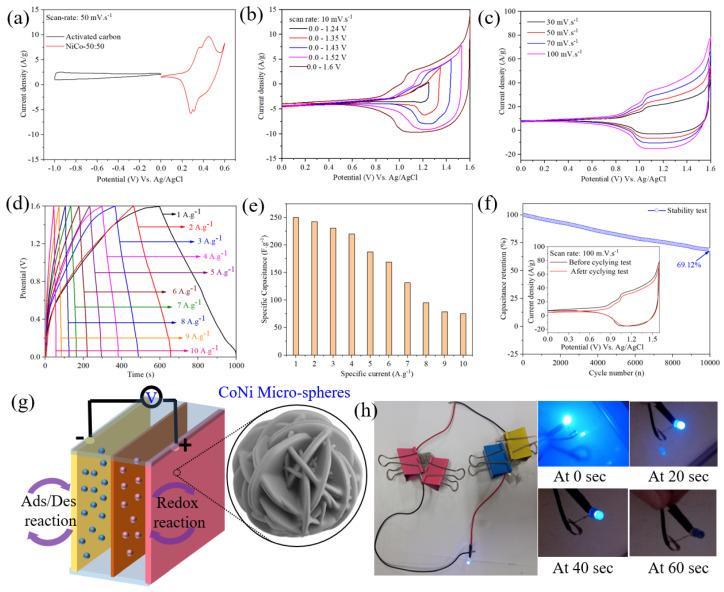
(**a**) CVs of NiCo-75:25 electrode and AC electrode. (**b**) Extended voltage window stability of the ASC. (**c**) CVs of the ASC at different sweep rates. (**d**) GCD profile of the ASC at various current densities. (**e**) Specific capacitance (Cs) as a function of current density. (**f**) Cycling stability of the ASC with consistent CV analysis. (**g**) Schematic diagram of the assembled ASC device. (**h**) Real-world test demonstrating LED illumination powered by two ASCs.

The ASC configuration integrates the NiCo-75:25 electrode material as the positrode and activated carbon (AC) electrodes as the negatrode, as illustrated in Figure 4. Combining a Faradic redox electrode (NiCo-75:25) with an EDLC-type electrode (AC), the ASC facilitates a redox reaction where cation (OH^−^) and anion (K^+^) adsorption and desorption occur at the positrode and negatrode, respectively [60]. AC was chosen for its high specific surface area, excellent electrical conductivity, and good electrochemical stability. This choice facilitates the formation of a larger number of double layers, facilitating the transport of electrolyte ions and enhancing energy storage capacity and rate capability. Balancing the mass between positrode and negatrode is crucial for achieving higher supercapacitive performance and better energy efficiency before assembling the ASC. The optimal NiCo-75:25-to-AC mass ratio was approximately 1:1, with an overall weight of about 3.1 mg cm^−2^. The stable working voltage window of the ASC was determined based on the CVs of the NiCo-75:25 electrode (0–0.6 V) and AC electrode (−1.0 to 0 V) performed at a sweep rate of 50 mV s^−1^, as depicted in Figure 4a. The plausible Faradic reactions that take place in the NiCo-75:25-based BTMOCs electrode surface from the combined redox behavior of Co and Ni^+^ ions could be described as follows [61]:

(5) $${Ni}^{+}+{3OH}^{-}↔NiOOH+H_{2}O+2e^{-}$$

(6) $${Ni}^{2+}+{3OH}^{-}↔NiOOH+H_{2}O+e^{-}$$

(7) $$NiOOH+{OH}^{-}↔{NiO}_{2}+H_{2}O+e^{-}$$

(8) $${Co}^{2+}+{3OH}^{-}↔CoOOH+H_{2}O+e^{-}$$

(9) $${Co}^{3+}+{3OH}^{-}↔CoOOH+H_{2}O$$

(10) $$CoOOH+{OH}^{-}↔{CoO}_{2}+{2H}_{2}O+e^{-}$$

Meanwhile, for the AC electrode, its CV curve indicates the capacitive behavior of the electrode where the charges are stored as electric double layer (EDL), and it is designated as follows: AC + xOH^−^ + yK^+^ ↔ AC||xOH^−^ + yK^+^, where || indicates the double-layer formation.

Notably, the stable operating window of the ASC extends to 1.6 V (Figure 4b). The CVs of the ASC at different sweep rates within the voltage window of 0–1.6 V (Figure 4c) demonstrate preserved CV shapes even at a sweep rate of 100 mV s^−1^, indicating a good rate performance and fast charge/discharge behavior [62]. The GCD profile of the ASC at various current densities (1–10 A g^−1^) within the voltage window of 0–1.6 V is shown in Figure 4d, with symmetrical profiles indicating excellent electrochemical reversibility [62].

The C_s_ of the ASC, determined as a function of current density (Figure 4e), is estimated at 250 F g^−1^ at a current density of 1 A g^−1^. Remarkably, the ASC retains 74% and 30% of its initial capacitance even at current densities of 5 A g^−1^ and 10 A g^−1^, respectively, demonstrating high-rate capability [63]. The ED and PD of the ASC, estimated using Equations (3) and (4), are found to be 88.8 Wh Kg^−1^ and 800 W Kg^−1^, respectively, showcasing its exceptional performance. Additionally, the cyclic lifetime of the ASC, evaluated at a current density of 1 A g^−1^, demonstrates long-term cycling retention with less than 69% deterioration of its initial Cs over 10,000 continuous GCD cycles (Figure 4f). The consistent CV analysis of the (inset plot in Figure 4f) pre- and post-cyclic stability testing underscores the remarkable stability of the electrode material, affirming the exceptional electrochemical performance of NiCo-75:25 as a viable candidate for ASC applications. However, despite the enhanced electrochemical performance and stability attributed to the excellent structural characteristics of the BTMOCs, such as their 3D morphology and mesoporous sheet-like architecture, it is noteworthy that the retention capacitance was not exceptionally high (69%). The observed degradation in capacity, beginning after the initial ten cycles and persisting consistently throughout the 10,000 cycles, suggests the formation of a solid electrolyte interface (SEI) layer on the surface of the BTMOCs during each charge and discharge cycle. This SEI layer, estimated to be a few nanometers thick, likely impedes ion accessibility, resulting in reduced capacitance. Furthermore, the electrochemical performance of the fabricated ASC is compared relatively with recent reports, as shown in Table 1. To assess the practical applicability of the fabricated electrode for energy storage, a real-world test was conducted using a commercial LED powered by two assembled ASCs (NiCo-75:25//AC). To illustrate the setup, Figure 4g depicts the schematic diagram of the assembled ASC device. After the ASC devices were charged with a 9 V battery, they were promptly connected in series to power the blue LED for approximately 60 s, as presented in Figure 4h. Notably, the LED’s luminosity gradually decreased from 20 s to 40 s, and beyond 60 s, the brightness diminished significantly. This practical demonstration underscores the potential efficiency and suitability of the fabricated ASC for various energy storage applications. However, a slight flaw in the demonstration was observed as the LED’s luminosity diminished beyond 60 s, suggesting a limitation in prolonged power supply.

## 4. Conclusions

In conclusion, our study presents a facile method for synthesizing layered NiCo-75:25 BTMOCs, presenting remarkable potential for high-performance supercapacitor applications. The NiCo-75:25 BTMOCs exhibit a unique hierarchical nanostructure with flower-shaped microspheres, resulting in a notably high specific surface area of 23.66 m^2^ g^−1^. This enhancement facilitates efficient redox reactions and offers promising electrochemical properties. The NiCo-75:25-based electrode demonstrates an impressive specific capacitance of 888.8 F g^−1^ at a specific current of 1 A g^−1^, presenting its suitability for high-rate applications. Moreover, the assembled ASC comprising NiCo-75:25 as the positive electrode and AC as the negative electrode achieves a specific capacitance of 250 F g^−1^ at a specific current of 1 A g^−1^. Additionally, the ASC exhibits a high energy density of 88 Wh Kg^−1^ at a power density of 800 W kg^−1^ and excellent long-term cyclability with >69% retention over 10,000 charge–discharge cycles. These results underscore the importance of optimizing the NiCo ratio in BTMOCs to achieve superior supercapacitor performance. Future research directions may focus on further optimizing the synthesis process and exploring practical applications of NiCo-75:25-based supercapacitors in energy storage systems.

## Figures and Tables

**Table 1:** Comparing the asymmetric supercapacitors’ performance with other previous systems.

Materials	Current Density (A/g)	C_sp_ (F/g)	E_D_ (Wh/kg)	P_D_ (W/kg)	Ref.
ZnO–NiO–CuO mixed metal oxides	1	118	15.7	-	[64]
Mesoporous NiO nanosheets	2	202.3	47.18	758.37	[65]
CoO@CuONanowire arrays	1	56.88	20.22	800	[66]
CoO@CoAl-LDH hierarchical 3D nanobouquet arrays	1	195.6	69.55	800	[67]
Ni-Co metal oxides	1	484.4	50.3	300.2	[68]
Tri-metallic MOF nanoarchitecture	1	166.4	23.6	501.5	[69]
CuCo_2_O_4_/CuO//AC	1	115	28.12	874.84	[70]
NiCo-75:25//AC	1	250	88	800	This study

## Data Availability

The data presented in the study are available from the corresponding author.

## References

[1] Gao M., Wang Z., Liu Z., Huang Y., Wang F., Wang M., Yang S., Li J., Liu J., Qi H. (2023). 2D Conjugated Metal–Organic Frameworks Embedded with Iodine for High-Performance Ammonium-Ion Hybrid Supercapacitors. Adv. Mater..

[2] Zhu X., Zhang Y., Man Z., Lu W., Chen W., Xu J., Bao N., Chen W., Wu G. (2023). Microfluidic-Assembled Covalent Organic Frameworks@ Ti3C2Tx MXene Vertical Fibers for High-Performance Electrochemical Supercapacitors. Adv. Mater..

[3] Pour G.B., Fard H.N., Aval L.F., Dubal D. (2023). Recent advances in Ni-materials/carbon nanocomposites for supercapacitor electrodes. Mater. Adv..

[4] Deshagani S., Maity D., Das A., Deepa M. (2021). NiMoO_4_@ NiMnCo_2_O_4_ heterostructure: A poly (3, 4-propylenedioxythiophene) composite-based supercapacitor powers an electrochromic device. ACS Appl. Mater. Interfaces.

[5] Yu J., Su C., Shang L., Zhang T. (2023). Single-Atom-Based Oxygen Reduction Reaction Catalysts for Proton Exchange Membrane Fuel Cells: Progress and Perspective. ACS Nano.

[6] Minakshi M., Schneider P.A., Ahuja R. (2021). 10 suitable electrode materials for hybrid capacitors. Next-Generation Materials for Batteries.

[7] He H., Si J., Zeng S., Ren N., Liu H., Chen C.-H. (2023). Vanadium-Based Pyrophosphate Material K_2_(VO)_3_(P_2_O_7_)_2_ as a High Voltage Cathode for Potassium Ion Batteries. ACS Appl. Energy Mater..

[8] Li Y., Shi J., Wu F., Li Y., Feng X., Liu M., Wu C., Bai Y. (2024). Dual-Functionalized Ca Enables High Sodiation Kinetics for Hard Carbon in Sodium-Ion Batteries. ACS Appl. Mater. Interfaces.

[9] Sheng O., Jin C., Yang T., Ju Z., Luo J., Tao X. (2023). Designing biomass-integrated solid polymer electrolytes for safe and energy-dense lithium metal batteries. Energy Environ. Sci..

[10] Lv S., Fang T., Ding Z., Wang Y., Jiang H., Wei C., Zhou D., Tang X., Liu X. (2022). A High-Performance Quasi-Solid-State Aqueous Zinc–Dual Halogen Battery. ACS Nano.

[11] Yu Y., Li M., Zhou J., Sun M., Sun X., Jiang Z., Li Y., Wang C. (2024). Structural designs of advanced wood-based thick electrodes for high-performance eco-supercapacitors. Nano Today.

[12] Wang Y., Wei Z., Ji T., Bai R., Zhu H. (2023). Highly Ionic Conductive, Stretchable, and Tough Ionogel for Flexible Solid-State Supercapacitor. Small.

[13] Shaheen I., Hussain I., Zahra T., Javed M.S., Shah S.S.A., Khan K., Hanif M.B., Assiri M.A., Said Z., Arifeen W.U. (2023). Recent advancements in metal oxides for energy storage materials: Design, classification, and electrodes configuration of supercapacitor. J. Energy Storage.

[14] Cheng Y., Xia K., Li H., Liu P., Zhao Z., Xu G., Wahid F., Wang H. (2023). One-pot synthesis of NiO-MnCo_2_O_4_ heterostructure hollow spheres via template-free solvothermal method for high-performance supercapacitors. Colloids Surf. A Physicochem. Eng. Asp..

[15] Prabhu R., Jayarambabu N., Anitha N., Jitesh P., Hitesh B., Rao T.V. (2024). Size dependent electrochemical properties of green synthesized NiO nanoparticles as a supercapacitor electrode. Inorg. Chem. Commun..

[16] Ramkumar R., Minakshi M. (2015). Fabrication of ultrathin CoMoO_4_ nanosheets modified with chitosan and their improved performance in energy storage device. Dalton Trans..

[17] Sharma P., Minakshi Sundaram M., Watcharatharapong T., Laird D., Euchner H., Ahuja R. (2020). Zn metal atom doping on the surface plane of one-dimesional NiMoO_4_ nanorods with improved redox chemistry. ACS Appl. Mater. Interfaces.

[18] Zhou H., Wang X.-L., Nasser R., Jiang T.-T., Li Z., Song J.-M. (2024). Flower-like FeCoNi ternary composite formed by interweaving nano needles for positive electrode material of supercapacitor. J. Alloys Compd..

[19] Raveendran A., Chandran M., Siddiqui M.R., Wabaidur S.M., Angaiah S., Dhanusuraman R. (2024). Binary Ni–Cu nanocomposite-modified MXene-adorned 3D-nickel foam for effective overall water splitting and supercapacitor applications. Sustain. Energy Fuels.

[20] Gupta M., Tyagi S., Kumari N. (2024). Electrochemical performance of hybrid spinel ferrite/carbon (NiFe_2_O_4_/C) nanocomposite derived from metal organic frameworks (MOF) as electrode material for supercapacitor application. J. Solid State Electrochem..

[21] Yue L., Chen L., Wang X., Lu D., Zhou W., Shen D., Yang Q., Xiao S., Li Y. (2023). Ni/Co-MOF@ aminated MXene hierarchical electrodes for high-stability supercapacitors. Chem. Eng. J..

[22] Xu D., Zhang Z., Tao K., Han L. (2023). A heterostructure of a 2D bimetallic metal–organic framework assembled on an MXene for high-performance supercapacitors. Dalton Trans..

[23] Xi Y., Yan J., An B. (2023). Kinetic study of hybrid supercapacitor using transition metal quantum dots@ graphenes composite as a model electrode. Appl. Surf. Sci..

[24] Wu H., Li S., Liu Y., Mu Q., Shi Y. (2024). Dual metal MOF derived Co-Ni/rGO as cathode material with synergetic effect for an asymmetric supercapacitor with enhanced performances. J. Energy Storage.

[25] Hilal M., Xie W., Yang W. (2022). Straw-sheaf–like Co_3_O_4_ for preparation of an electrochemical non-enzymatic glucose sensor. Microchim. Acta.

[26] Hilal M., Lee S., Hwang Y. (2023). Facial preparation of electrochemically stable NiO/ZnO hybrid electrode exhibiting excellent redox activity for enhanced glucose detection. Surf. Interfaces.

[27] Zhang W., Jia G., Li H., Liu S., Yuan C., Bai Y., Fu D. (2016). Morphology-modulated mesoporous CuO electrodes for efficient interfacial contact in nonenzymatic glucose sensors and high-performance supercapacitors. J. Electrochem. Soc..

[28] Li Z., Shao M., Zhou L., Zhang R., Zhang C., Han J., Wei M., Evans D.G., Duan X. (2016). A flexible all-solid-state micro-supercapacitor based on hierarchical CuO@ layered double hydroxide core–shell nanoarrays. Nano Energy.

[29] Zhang D., Cao J., Zhang X., Insin N., Liu R., Qin J. (2020). NiMn layered double hydroxide nanosheets in-situ anchored on Ti_3_C_2_ MXene via chemical bonds for superior supercapacitors. ACS Appl. Energy Mater..

[30] Gowdhaman A., Kumar S.A., Elumalai D., Balaji C., Sabarinathan M., Ramesh R., Navaneethan M. (2023). Ni-MOF derived NiO/Ni/r-GO nanocomposite as a novel electrode material for high-performance asymmetric supercapacitor. J. Energy Storage.

[31] Jia J., Qin Z., Yang X., Peng X., Ren G., Lin Z. (2023). SiO_2_ anchored stacked-petal structure CoO-NiO/CNF as electrodes for high-rate-performance supercapacitors. Diam. Relat. Mater..

[32] Biswal A., Panda P., Jiang Z.-T., Tripathy B., Minakshi M. (2019). Facile synthesis of a nanoporous sea sponge architecture in a binary metal oxide. Nanoscale Adv..

[33] Ragab H., Diab N., Khaled A.M., Al Ojeery A., Al-Hakimi A.N., Farea M. (2024). Incorporating hybrid Ag/Co_2_O_3_ nanofillers into PVP/CS blends for multifunctional optoelectronic and nanodielectric applications. Ceram. Int..

[34] Rabia M., Essam D., Alkallas F.H., Shaban M., Elaissi S., Ben Gouider Trabelsi A. (2022). Flower-shaped CoS-Co_2_O_3_/G-C_3_N_4_ nanocomposite for two-symmetric-electrodes supercapacitor of high capacitance efficiency examined in basic and acidic mediums. Micromachines.

[35] He H., Feng J., Gao X., Fei X. (2022). Selective separation and recovery of lithium, nickel, MnO_2_, and Co_2_O_3_ from LiNi_0.5_Mn_0.3_Co_0.2_O_2_ in spent battery. Chemosphere.

[36] Samal R., Dash B., Sarangi C.K., Sanjay K., Subbaiah T., Senanayake G., Minakshi M. (2017). Influence of synthesis temperature on the growth and surface morphology of Co3O_4_ nanocubes for supercapacitor applications. Nanomaterials.

[37] Iftikhar M., Latif S., Jevtovic V., Ashraf I., El-Zahhar A.A., Saleh E.A.M., Abbas S.M. (2022). Current advances and prospects in NiO-based lithium-ion battery anodes. Sustain. Energy Technol. Assess..

[38] Pu J., Wang T., Zhu X., Tan Y., Gao L., Chen J., Huang J., Wang Z. (2022). Multifunctional Ni/NiO heterostructure nanoparticles doped carbon nanorods modified separator for enhancing Li–S battery performance. Electrochim. Acta.

[39] Liu S., Zeng W., Chen T. (2017). Synthesis of hierarchical flower-like NiO and the influence of surfactant. Phys. E Low-Dimens. Syst. Nanostructures.

[40] Parveen N., Cho M.H. (2016). Self-assembled 3D flower-like nickel hydroxide nanostructures and their supercapacitor applications. Sci. Rep..

[41] Velhal N.B., Yun T.H., Ahn J., Kim T., Kim J., Yim C. (2023). Tailoring cobalt oxide nanostructures for stable and high-performance energy storage applications. Ceram. Int..

[42] Ghaedi H., Zhao M. (2022). Review on template removal techniques for synthesis of mesoporous silica materials. Energy Fuels.

[43] Cui Z., Wang T., Geng Z., Wan L., Liu Y., Xu S., Gao N., Li H., Yang M. (2024). CoNiO_2_/Co_3_O_4_ Nanosheets on Boron Doped Diamond for Supercapacitor Electrodes. Nanomaterials.

[44] Wang Z., Lian Y., Zhu X., Wang Q. (2024). MOF-Mediated Construction of NiCoMn-LDH Nanoflakes Assembled Co (OH) F Nanorods for Improved Supercapacitive Performance. Nanomaterials.

[45] Zhang J., Lu H., Yao T., Ji X., Zhang Q., Meng L., Feng J., Wang H. (2024). Copper-induced formation of heterostructured Co_3_O_4_/CuO hollow nanospheres towards greatly enhanced lithium storage performance. Chin. Chem. Lett..

[46] Pai S.H.S., Sasmal A., Nayak A.K., Han H. (2023). Facile Solvothermal Synthesis of NiO/gC_3_N_4_ Nanocomposite for Enhanced Supercapacitor Application. Int. J. Energy Res..

[47] Hilal M., Yang W., Hwang Y., Xie W. (2024). Tailoring MXene Thickness and Functionalization for Enhanced Room-Temperature Trace NO_2_ Sensing. Nano-Micro Lett..

[48] Rajasekhar D., Naresh B., Madhavi V., Krishna K.G., Kuchi C., Kumar K.S., Reddy P.S. (2023). Hierarchical NiCo_2_O_4_/NiO mixed nanofibers for enhanced supercapacitor and ammonia gas sensor applications. Inorg. Chem. Commun..

[49] Idriss H. (2021). On the wrong assignment of the XPS O1s signal at 531–532 eV attributed to oxygen vacancies in photo-and electro-catalysts for water splitting and other materials applications. Surf. Sci..

[50] Hilal M., Han J.I. (2018). Significant improvement in the photovoltaic stability of bulk heterojunction organic solar cells by the molecular level interaction of graphene oxide with a PEDOT: PSS composite hole transport layer. Sol. Energy.

[51] Parveen N., Hilal M., Han J.I. (2020). Newly design porous/sponge red phosphorus@ graphene and highly conductive Ni_2_P electrode for asymmetric solid state supercapacitive device with excellent performance. Nano-Micro Lett..

[52] Kandasamy N., Venugopal T., Kannan K. (2018). Facile one-pot synthesis of flower like cobalt oxide nanostructures on nickel plate and its supercapacitance properties. J. Nanosci. Nanotechnol..

[53] Sivakumar P., Vikraman D., Raj C.J., Hussain S., Park J., Kim H.S., Jung H. (2021). Hierarchical NiCo/NiO/NiCo_2_O_4_ composite formation by solvothermal reaction as a potential electrode material for hydrogen evolutions and asymmetric supercapacitors. Int. J. Energy Res..

[54] Chatterjee S., Maiti R., Miah M., Saha S.K., Chakravorty D. (2017). NiO nanoparticle synthesis using a triblock copolymer: Enhanced magnetization and high specific capacitance of electrodes prepared from the powder. ACS Omega.

[55] Sivakumar P., Raj C.J., Jung H., Park H.S. (2023). 2D/2D nanoarchitecture of Ni/NiCo_2_O_4_ deposited onto reduced graphene oxide for high-performance hybrid supercapacitor applications. J. Energy Storage.

[56] Karuppasamy K., Vikraman D., Hussain S., Santhoshkumar P., Bose R., Sivakumar P., Alfantazi A., Jung J., Kim H.S. (2022). Unveiling the Redox Electrochemistry of MOF-Derived fcc-NiCo@ GC Polyhedron as an Advanced Electrode Material for Boosting Specific Energy of the Supercapattery. Small.

[57] Xiong L.-Y., Kim Y.-J., Seo W.-C., Lee H.-K., Yang W.-C., Xie W.-F. (2023). High-performance non-enzymatic glucose sensor based on Co_3_O_4_/rGO nanohybrid. Rare Met..

[58] Wang C., Sui G., Guo D., Li J., Ma X., Zhuang Y., Chai D.-F. (2022). Oxygen vacancies-rich NiCo_2_O_4-4x_ nanowires assembled on porous carbon derived from cigarette ash: A competitive candidate for hydrogen evolution reaction and supercapacitor. J. Energy Storage.

[59] Mule A.R., Ramulu B., Arbaz S.J., Kurakula A., Yu J.S. (2024). Ag-integrated mixed metallic Co-Fe-Ni-Mn hydroxide composite as advanced electrode for high-performance hybrid supercapacitors. J. Energy Chem..

[60] Majumdar D., Mandal M., Bhattacharya S.K. (2020). Journey from supercapacitors to supercapatteries: Recent advancements in electrochemical energy storage systems. Emergent Mater..

[61] Savariraj A.D., Raj C.J., Velayutham R., Jang H.M., Sivakumar P., Cho W.-J., Kim B.C. (2023). Experimental and theoretical exploration of nickel ion-implanted metal-organic frameworks (ZIF-67) as free-standing electrodes for hybrid supercapacitors. J. Power Sources.

[62] Ghaly H.A., El-Deen A.G., Souaya E.R., Allam N.K. (2019). Asymmetric supercapacitors based on 3D graphene-wrapped V_2_O_5_ nanospheres and Fe_3_O_4_@ 3D graphene electrodes with high power and energy densities. Electrochim. Acta.

[63] Rathore H.K., Hariram M., Ganesha M.K., Singh A.K., Das D., Kumar M., Awasthi K., Sarkar D. (2023). Understanding supercapacitive performance of a N-doped vanadium carbide/carbon composite as an anode material in an all pseudocapacitive asymmetric cell. Sustain. Energy Fuels.

[64] Packiaraj R., Mahendraprabhu K., Devendran P., Nallamuthu N., Palanivel B., Venkatesh K., Karuppannan R. (2021). Electrochemical performances of ZnO–NiO–CuO mixed metal oxides as smart electrode material for solid-state asymmetric device fabrication. Energy Fuels.

[65] Ambade R.B., Lee H., Lee K.H., Lee H., Veerasubramani G.K., Kim Y.-B., Han T.H. (2022). Ultrafast flashlight sintered mesoporous NiO nanosheets for stable asymmetric supercapacitors. Chem. Eng. J..

[66] Li X., Huang W., Zhong Y., Liao L., Cheng Y., Zheng K., Liu J. (2022). Dandelion-like Nanospheres Synthesized by CoO@ CuO Nanowire Arrays for High-Performance Asymmetric Supercapacitors. ChemElectroChem.

[67] Song Y., Shen Q., Pan G.-X., Ye C., Zhang Y.-F., Song L. (2023). A high-performance asymmetric supercapacitor device based on CoO@ CoAl-LDH hierarchical 3D nanobouquet arrays. CrystEngComm.

[68] Zhang X., Javed M.S., Zhang X., Ali S., Han K., Ahmad A., Hussain I., Tighezza A.M., Arifeen W.U., Han W. (2024). Synergistic effects of defects and surface engineering in Ni-Co metal oxides to improve the high performance of Zn-ion hybrid supercapacitors. J. Energy Storage.

[69] Anwer A.H., Ansari M.Z., Mashkoor F., Zhu S., Shoeb M., Jeong C. (2023). Synergistic effect of carbon nanotube and tri-metallic MOF nanoarchitecture for electrochemical high-performance asymmetric supercapacitor applications and their charge storage mechanism. J. Alloys Compd..

[70] Mao H., Sun J., Bao E., Dai L., Xu C., Chen H. (2021). Battery-type CuCo_2_O_4_/CuO nanocomposites as positive electrode materials for highly capable hybrid supercapacitors. Ceram. Int..

